# The effect of pre-incubation of *Allium cepa* L. roots in the ATH-rich extract on Pb uptake and localization

**DOI:** 10.1007/s00709-012-0445-z

**Published:** 2012-08-16

**Authors:** Sława Glińska, Magdalena Gapińska

**Affiliations:** Laboratory of Electron Microscopy, Faculty of Biology and Environmental Protection, University of Lodz, Banacha 12/16, 90-237 Lodz, Poland

**Keywords:** *Allium cepa*, Anthocyanins, Lead localization, Ultrastructure

## Abstract

The positive influence of anthocyanin (ATH) on toxic metal-treated plant material is well documented; however, it is still not explained if it is caused by changes in element absorption and distribution. Therefore, detailed analysis of the effect of the ATH-rich extract from red cabbage leaves on Pb uptake and localization at morphological, anatomical and ultrastructural level was the goal of this study. Two-day-old adventitious roots of *Allium cepa* L. (cv. Polanowska) were treated for 2 h with the aqueous solution of Pb(NO_3_)_2_ at the concentration of 100 μM with or without preliminary incubation in the anthocyanin-rich extract from *Brassica oleracea* L. var. *capitata rubra* leaves (250 μM, 3 h). The red cabbage extract did not change the total Pb uptake but it enhanced the translocation of accumulated metal from roots to shoots. Within the pretreated roots, more Pb was deposited in their basal part and definitely smaller amount of the metal was bound in the apoplast of the outer layers of cortex cells. The ultrastructural analysis (transmission electron microscopy and X-ray microanalysis) revealed that the ATH-rich extract lowered the number of Pb deposits in intracellular spaces, cell wall and cytoplasm of root meristematic cells as well as in such organelles important to cell metabolism as mitochondria, plastids and nucleus. The Pb deposits were preferably localised in those vacuoles where ATH also occurred. This sequestration of Pb in vacuoles is probably responsible for reduction of metal cytotoxicity and consequently could lead to better plant growth.

## Introduction

Human activity has led to high level of Pb being accumulated from sewage sludge or urban composts, industrial effluents, household chimneys, emissions from municipal waste incinerators, fertilisers and pesticides or herbicides, residues from mining and the metal smelting industry (Brännvall et al. 1999; Buchauer 1973; Douay et al. 2008; Lagerwer and Specht 1970; Nriagu and Pacyna 1988). High contamination of the environment with heavy metals is still a running issue in Europe (HELCOM 2007; Suciu et al. 2008). Poland is the country with the highest contribution to annual deposition of those toxic elements to the Baltic Sea (HELCOM 2007). Accumulation of Pb, a non-essential toxic element, in the atmosphere, water and soil can be dangerous to all kinds of organisms including human beings (Douay et al. 2008; Velea et al. 2009). Studies on Pb effect on plants reveal that this metal is strongly phytotoxic and causes growth inhibition, genotoxicity and even plant death (Gichner et al. 2008; Liu et al. 2008). Therefore, high contamination of some industrialised regions with Pb and other heavy metals triggered continuous biological studies aiming of elucidating the mechanisms of transport and deposition of these pollutants in plants as well as strategies of developing resistance to this kind of stress factors. One of the methods of minimising the toxic effect of metals is chelating them and removing from an organism or sequestrating in separated cell compartments (Hale et al. 2001; Zenk 1996). It is known that flavonoids besides their antioxidant properties could act as natural chelators because they are able to form strong ligand complexes with ions of minerals (Ferguson 2001; Hale et al. 2001; Havsteen 2002; Rice-Evans et al. 1997). Their chelating properties lead to metal isolation and sequestration, e.g. Mo in the epidermis of *Brassica* sp. (Hale et al. 2001).

The beneficial effects of anthocyanin (ATH)-rich extract from red cabbage leaves on animals were proved during in vivo and in vitro studies (Kolodziejczyk at al. 2011; Saluk et al. 2012; Sankhari et al. 2012). Moreover, in the last decade, the protective activity of ATH against heavy metal toxicity in animal and plant organisms was reported (Glińska et al. 2007; Kowalczyk et al. 2003). Experiments on rats showed that they reduced the harmful effects of Cd (Kowalczyk et al. 2003). Our earlier studies revealed the ability of the ATH-rich extract from red cabbage leaves to attenuate Cd, Cr and Pb toxicity, i.e. their mitodepressive and turbogenic effects in the root meristem of *Allium cepa* L. (Glińska et al. 2007). Moreover, the analysis of the same material at the ultrastructural level showed that the ATH-rich extract from red cabbage leaves did not disturb cell functions (Glińska and Gabara 2011) and lowered the number of nuclei with Pb deposits (Glińska et al. 2007). That effect might be connected with chelating properties of anthocyanins. Therefore, it would be interesting to examine whether the ATH-rich extract causes changes in metal absorption and deposition. The aim of the present work was to determine the effect of pre-incubation of *A. cepa* roots in ATH-rich extract from red cabbage leaves on Pb uptake and its localization at the morphological and ultrastructural levels.

## Materials and methods

### ATH extraction

Fresh leaves of red cabbage (*Brassica oleracea* var. *capitata rubra*) were extracted with the mixture of methanol/distilled water/0.01 % HCl (MeOH/H_2_O/HCl, 50/50/1, *v*/*v*/*w*) and centrifuged. The supernatant was dried in a vacuum rotary evaporator in water bath at 40 °C and than dissolved in distilled water pH 6.0. After centrifugation, ATH content was spectrophotometrically measured in the supernatant (Gitz et al. 1998). Total ATH concentration (in micromolars) was determined with cyanidin 3-glucoside as a standard and calculated using molecular density coefficient *ε* = 30 mM cm^−1^ at *λ* = 525 nm (Hodges and Nozzoillo 1996).

### Plant material and treatment

Healthy and equally sized bulbs of *A. cepa* L. (cv. Polanowska), obtained from ‘Polan’ Company (Cracow, Poland), after scale removing were placed in glass containers filled with Hoagland solution containing: KNO_3_ (0.51 g/l), Ca(NO_3_)_2_·4H_2_O (1.18 g/l), MgSO_4_·7H_2_O (1.23 g/l), KH_2_PO_4_ (0.14 g/l) and FeEDTA (5 mg/l) at pH 6.5, and cultured at 21 °C in darkness. The bulbs with 2-day-old adventitious roots were treated with the aqueous solution of Pb(NO_3_)_2_ at the concentration of 100 μM for 2 h with or without preliminary incubation in the ATH-rich extract (250 μM, 3 h). The roots kept in distilled water were the control. The used conditions were determined on the basis of preliminary experiments and are identical as in the previous papers (Glińska et al. 2007; Glińska and Gabara 2011).

### Determining the level of Pb in plant tissues

In order to determine the total amount of lead in roots and shoots, plant organs were dried at 60 °C until constant weight. The dried plant tissues (0.2 g) were put into Teflon vessels and 5 ml of 65 % HNO_3_ and 1 ml of 30 % H_2_O_2_ were added to each of them. The samples were digested in Ethos-1 microwave oven closed system (Milestone Inc.) at 200 °C for 20 min. After mineralisation, the samples were put to 25-ml measuring flasks which were filled with deionised water. Pb content was determined using Optima 2000 DV ICP-OES sequential spectrometer (Perkin-Elmer) at the wavelength 220.353 nm. The Merck ICP multi-element standard solution was used to prepare the calibration curve. The Pb content was calculated in milligrams per kilogram of dry weight (DW).

### Pb localization at morphological and anatomical level

Lead localisation in the whole roots and at cross sections was assayed using sodium rhodizonate method (Glińska and Gabara 2002). The roots were stained by soaking for 12 h in freshly prepared 0.2 % sodium rhodizonate in 0.1 M citrate buffer, pH 5.0. Then the excess of dye was washed away and the brown-red colouring of roots indicating the presence of Pb was analysed and documented. In order to establish lead localization at anatomical level, the handmade cross sections of the stained roots (0.5 cm from root tip) were analysed in light microscope ECLIPSE 50i (NICON) equipped with digital camera Power Shot A 640 (Canon).

### The sub-cellular localization of Pb

For determination of lead localisation at the ultrastructural level, root meristems (three per treatment) were fixed in 2 % glutaraldehyde in 0.1 M cacodylate buffer pH 7.5, for 2 h at 4 °C. Subsequently, they were rinsed with the same buffer and post-fixed in 1 % osmium tetroxide for 2 h at 4 °C. The material was dehydrated in a graded ethanol series and embedded in Epon-Spur’s resin mixture. Ultrathin sections were analysed before staining and again after staining in a saturated solution of uranyl acetate and with lead citrate (Reynolds 1963). Viewing of unstained sections permitted identification of lead deposits, as they are electron dense. To prove the presence of Pb in those deposits, the X-ray microanalysis of unstained ultrathin sections was performed using TEM JEM 1011 (JEOL) equipped with EDS INCA analyser (Oxford).

Subsequent staining of the same sections enabled ultrastructural analysis since the cell organelles became clearly visible. The cell ultrastructure in stained sections were examined in transmission electron microscope JEM 1010 (JEOL) at 80 kV. At least 60 microphotographs from each treatment were viewed.

### Statistical analysis

The experiment was triplicated. The results are presented as arithmetic means with standard error. All of the data and calculations were analysed by Microsoft Excel. Statistical significance of lead content was tested by Student’s *t* test for *α* < 0.05.

## Results

**Fig. 1:**
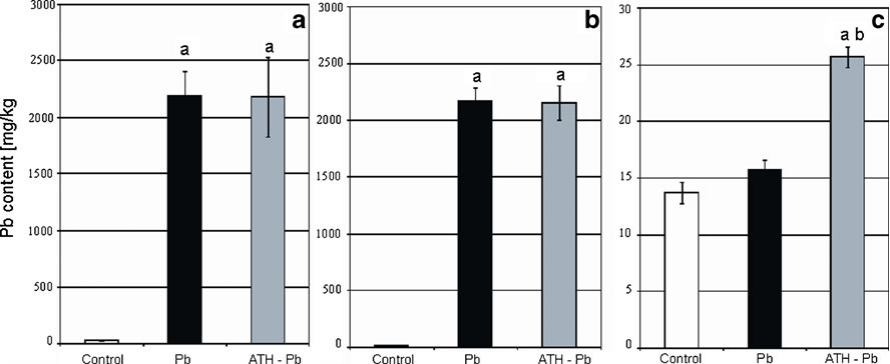
The effect of pre-incubation in the ATH-rich extract from red cabbage leaves on the concentration of Pb in a whole plant (**a**), roots (**b**) and shoot (**c**) of *A. cepa* growing in the presence of Pb(NO_3_)_2_. Values are means ± SE (*n* = 3). *Letters* denote statistically significant differences at the 0.05 level with Student’s *t* test between: the control and Pb-treated material (**a**), Pb-treated material with or without pre-incubation in the ATH-rich extract (**b**)

The ICP sequential spectrometry revealed that the Pb content in *A. cepa* plants incubated for 2 h in 100 μM lead nitrate solution dramatically increased in not pretreated as well as in ATH pretreated material (Fig. 1a). The lead-treated roots contained as much as 2,170 mg Pb/kg DW as compared with the control material that contained only 15 mg Pb/kg DW (Fig. 1b). Almost all Pb taken up by plants was accumulated in the roots, as the metal content in shoots did not significantly increase after Pb treatment as compared to the control (Fig. 1c). After ATH pretreatment, the amount of lead in the roots was not changed, but in the shoots, it increased by 63 % as compared to the material treated only with Pb(NO_3_)_2_ (Fig. 1b, c).

**Fig. 2:**
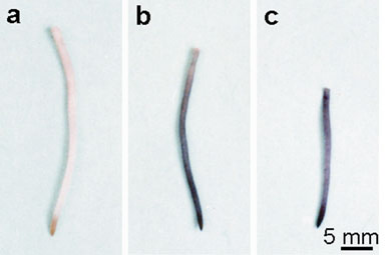
*A. cepa* roots stained with sodium rhodizonate. Brown colour indicates the presence of lead. **a** Control; **b** 100 μM Pb(NO_3_)_2_, 2 h; **c** 250 μM ATH-rich extract 3 h → 100 μM Pb(NO_3_)_2_, 2 h

**Fig. 3:**
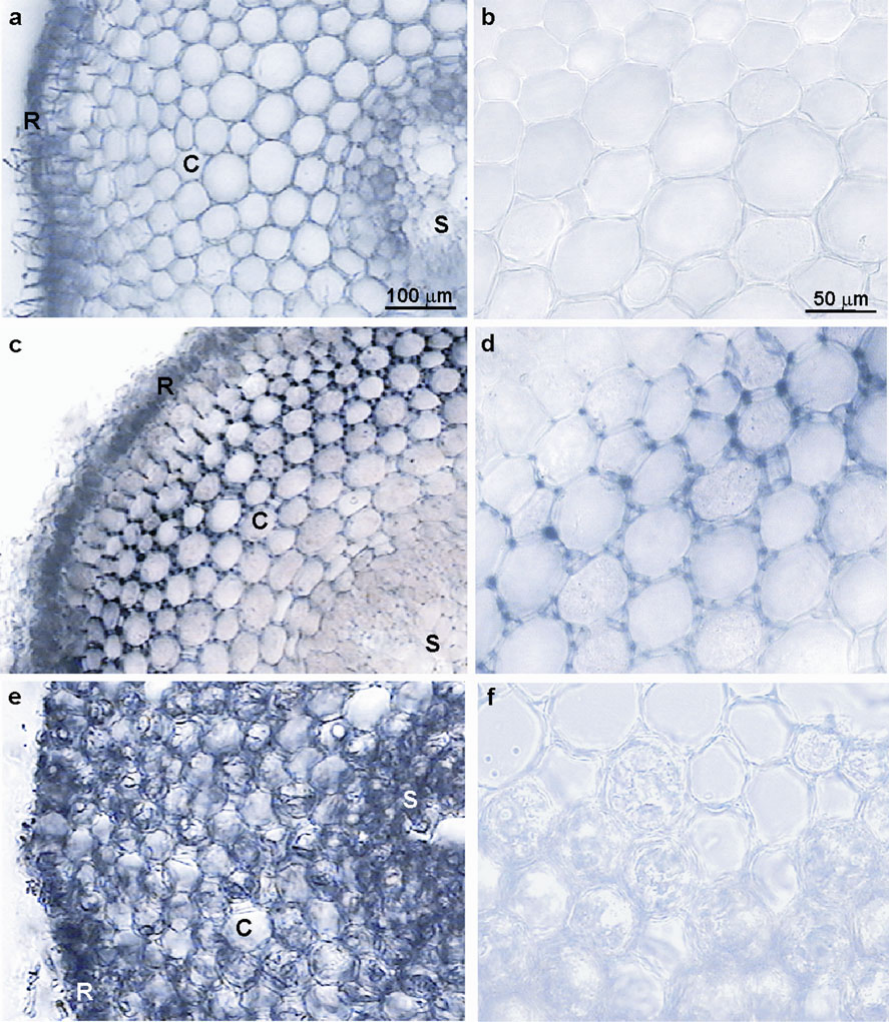
*A. cepa* roots stained with sodium rhodizonate—cross sections (0.5 cm from tip). **a**, **b** Control; **c**, **d** 100 μM Pb(NO_3_)_2_, 2 h; **e**, **f** 250 μM ATH-rich extract 3 h → 100 μM Pb(NO_3_)_2_, 2 h. **a**, **c**, **e** Differentiation zone of roots. **b**, **d**, **f** Cortex cells; *C* cortex, *R* rhizodermis, *S* stele

*A. cepa* roots treated with lead nitrate contained Pb as it was visualised by their brown colour after sodium rhodizonate staining, while the control roots remained unstained (Fig. 2a, b). Lead was not uniformly distributed along the root. The distal ends were most intensively stained and the colour intensity gradually decreased basipetally. In cross sections of the control roots, rhodizonate method did not reveal any Pb presence (Fig. 3a, b), but in cross sections of the differentiation zone of Pb(NO_3_)_2_-treated roots, large numerous lead deposits were localised in the rhizodermis and in the outer layers of cortex cells mainly in intercellular spaces and in cell walls (Fig. 3c, d). Small lead deposits were also present inside the cortex cells. Few lead deposits were visible in the apoplast of the inner cortex and in the stele (Fig. 3c, d).

In the roots pre-incubated in the ATH-rich extract and then treated with lead nitrate, sodium rhodizonate staining was less intensive in the differentiation zone but more visible in the basal part as compared to the roots only treated with lead (Fig. 2b, c). At the cross sections of those roots, lead occurred in the rhizodermis and its small deposits also within the vacuoles of cortex cells containing purple anthocyanins. The lead particles were not visible in intercellular spaces and cell walls (Fig. 3e, f).

**Fig. 4:**
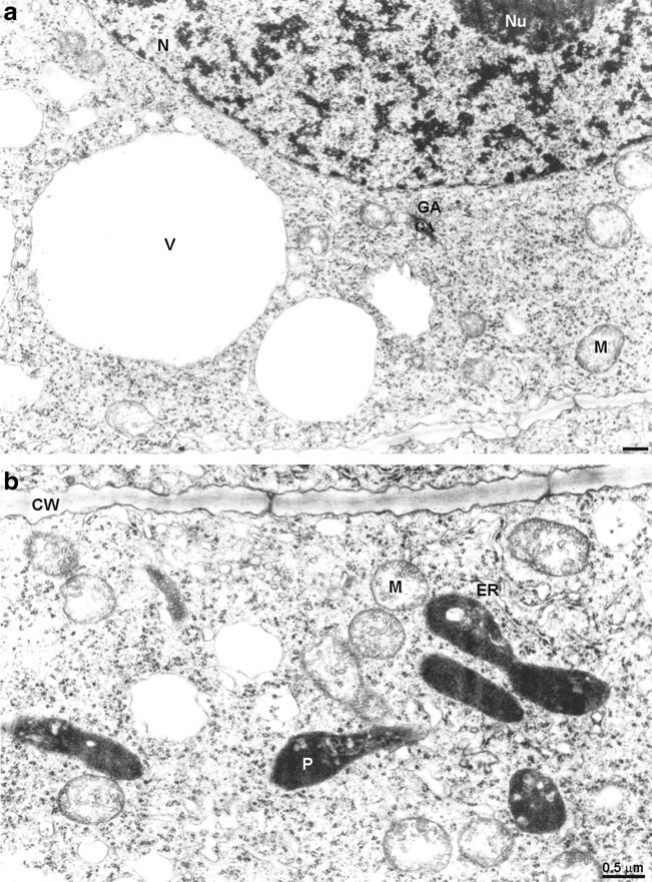
Meristematic cells of the control *A. cepa* root; **a**, **b** note lack of electron-dense deposits; *CW* cell wall, *ER* endoplasmic reticulum, *GA* Golgi apparatus, *M* mitochondrium, *N* nucleus, *Nu* nucleolus, *P* plastid, *V* vacuole

Ultrastructure of the control root meristematic cells was typical without any electron-dense deposits (Fig. 4a, b). A cell wall was thin and regular, a round nucleus contained a typical nucleolus, and in cytoplasm rich in ribosome, single endoplasmic reticulum cisternae were running in different directions (Fig. 4a, b). Golgi apparatus was composed of five to six cisternae and moderate number of vesicles (Fig. 4a). Moreover, in meristematic cells of the control roots, electron-transparent vacuoles occurred (Fig. 4a). Mitochondria displayed electron-transparent matrix and usually narrow cristae, plastids had electron-dense stroma and rare tylakoids (Fig. 4b).

**Fig. 5:**
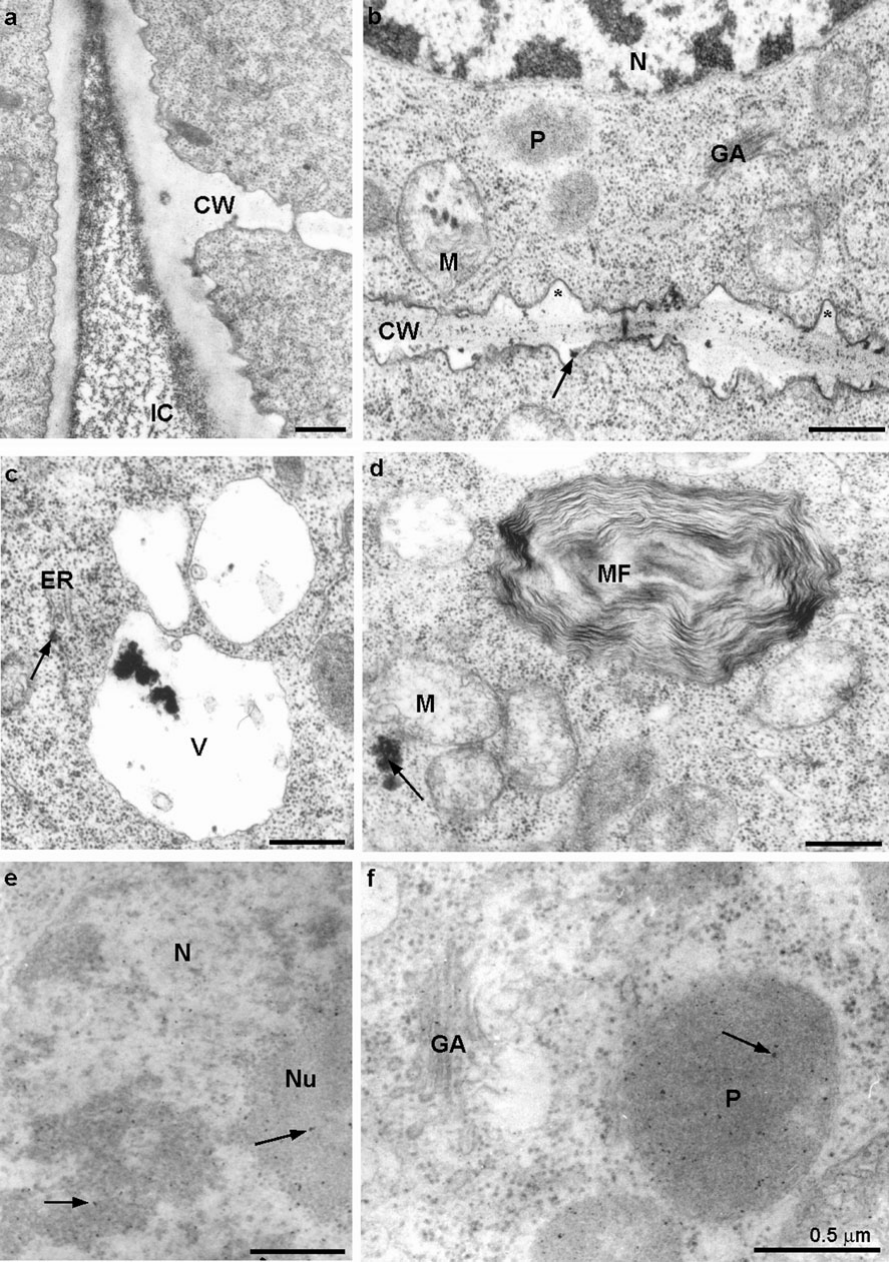
Meristematic cells of *A. cepa* roots treated with 100 μM Pb(NO_3_)_2_ for 2 h. **a** Numerous electron-dense deposits in the intercellular space; **b** numerous deposits in the cell wall (*arrow*) irregularly thickened (*asterisk*) and in mitochondria; **c** a small deposit in the lumen of ER (*arrow*), large ones in vacuole; **d** an electron-dense deposit in cytoplasm (*arrow*), myelinous figure visible; **e** small electron-dense deposits in nucleus and nucleolus (*arrows*); **f** small deposits in Golgi apparatus and in plastid (*arrow*); *CW* cell wall, *ER* endoplasmic reticulum, *GA* Golgi apparatus, *IC* intercellular space, *M* mitochondrium, *MF* myelinous figure, *N* nucleus, *Nu* nucleolus, *P* plastid, *V* vacuole

Pb(NO_3_)_2_-treated material revealed the presence of electron-dense dark deposits located mainly in the apoplast (Fig. 5a, b). Those deposits were observed most often in intercellular spaces (Fig. 5a), middle lamella and on the border between plasmalemma and a cell wall (Fig. 5b). Moreover, the cell wall was often locally thickened (Fig. 5b). Large electron-dense granules ware deposited in vacuoles (Fig. 5b). The presence of less numerous but sometimes quite big electron-dense deposits was also noticed in the cytoplasm (Fig. 5d). Small particles were present also in organelles such as plastids, mitochondria, Golgi apparatus and even in nuclei (Fig 5b, e, f). The electron-dense deposits were also sporadically seen in the cisternae of endoplasmic reticulum (Fig. 5c). Additionally in the cytoplasm of some cells, multilamellar structures appeared (Fig. 5d).

**Fig. 6:**
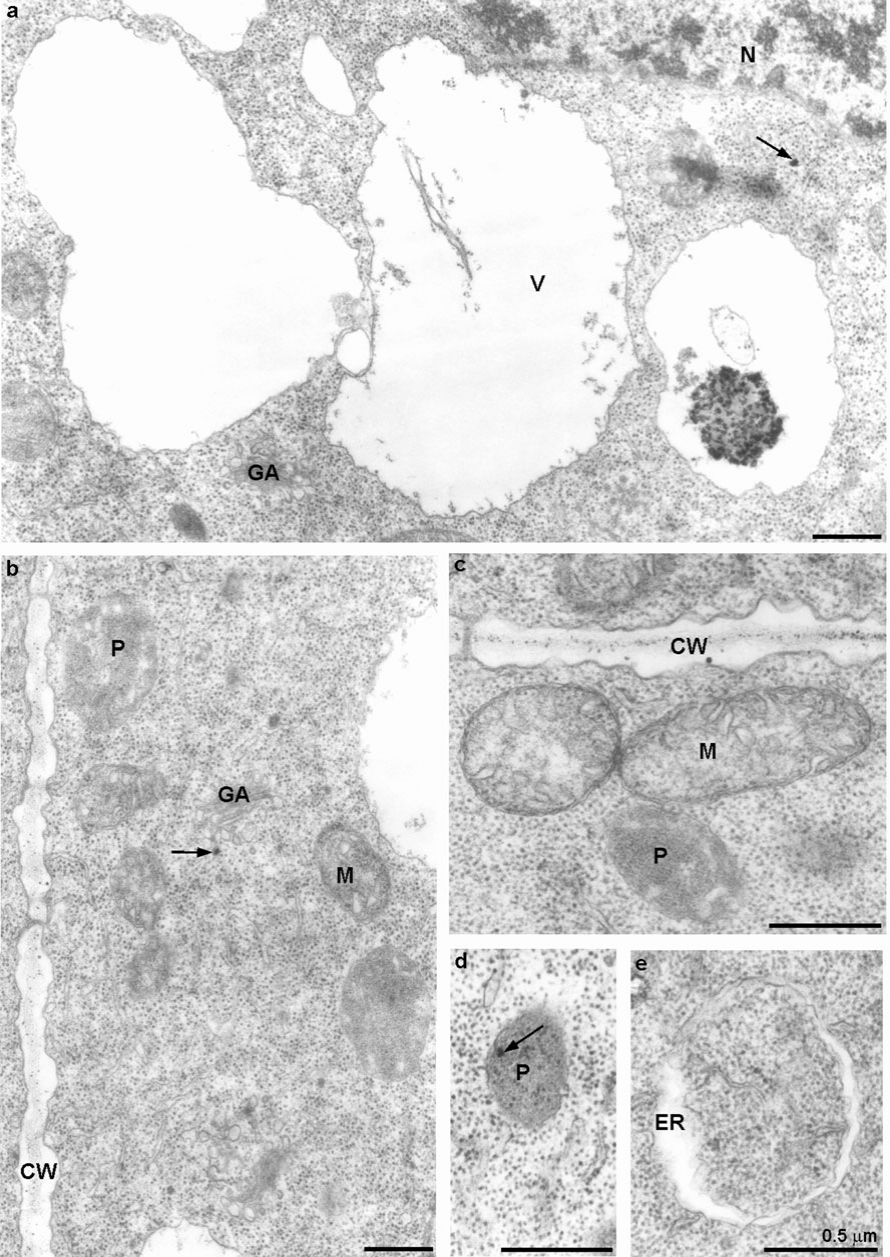
Meristematic cells of *A. cepa* roots pre-incubated with 250 μM ATH-rich extract for 3 h and subsequently treated with 100 μM Pb(NO_3_)_2_ for 2 h. **a** Large electron-dense deposits in the vacuole and a small one in cytoplasm (*arrow*); **b** very small deposits in the cell wall and vesicle of Golgi apparatus (*arrow*); **c** small electron-dense deposits in the cell wall as well as in mitochondria, and plastid deprived of deposits; **d** plastid with small electron-dense deposits (*arrow*); **e** swollen lumen of ER without deposits; *CW* cell wall, *ER* endoplasmic reticulum, *GA* Golgi apparatus, *M* mitochondrium, *N* nucleus, *P* plastid, *V* vacuole

The pre-incubation in the ATH-rich extract significantly diminished the number of electron-dense deposits in the apoplast (Fig. 6b, c). No dark particles were accumulated in intercellular spaces and apparently less deposits were visible in the cell walls which were thinner and less undulated than in material treated only with lead (Fig. 6b, c). Many vacuoles were electron-transparent and some contained dark granules assembled around an electron-dense grey material, presumably ATH (Fig. 6a). Small electron-dense deposits were sporadically noticed in the cytoplasm, Golgi apparatus and plastids (Fig. 6a, b, e). The pre-incubation of roots in the ATH-rich extract caused the disappearance of black deposits in the nuclei, mitochondria and ER (Fig. 6a, c, d). However, cisternae of ER became sporadically swollen and were occasionally circularly arranged (Fig. 6d).

**Fig. 7:**
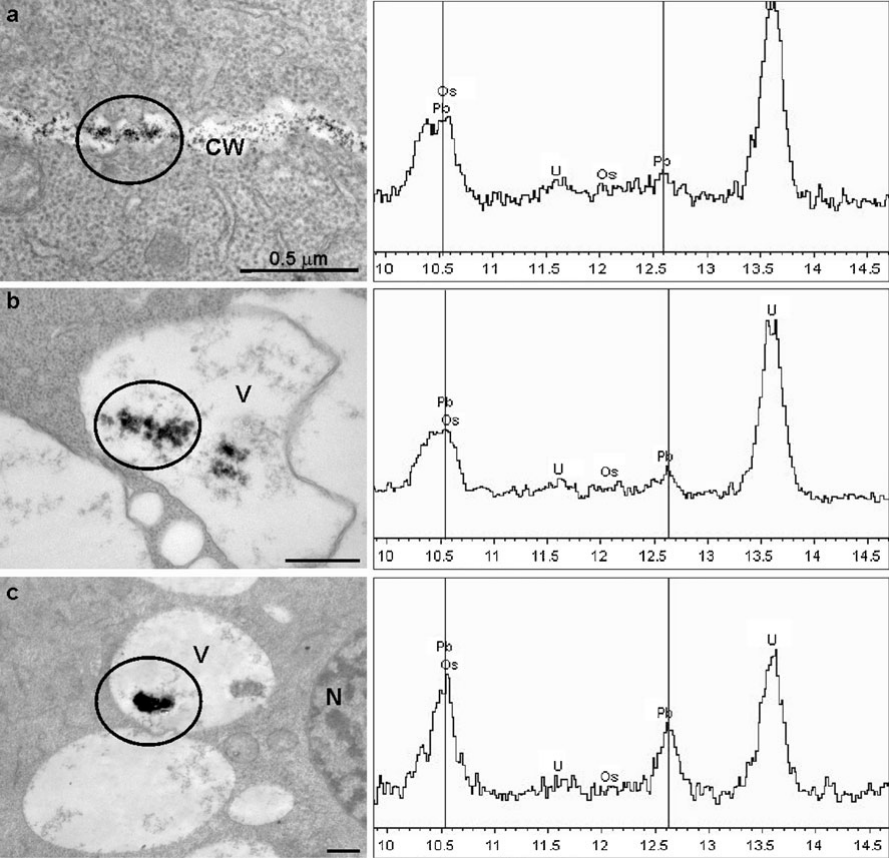
Electron micrographs illustrating distribution of electron-dense deposits in the root meristematic cells of *A. cepa* on unstained sections. The *circles* indicate the regions subjected to the X-ray microanalysis and spectra collected over those regions are presented on the *right*. The spectra (80 keV) show the L family of X-ray for Pb. The family consist of the *L*_α_ (10,540 keV) and *L*_β1_ (12,611 keV) peaks, which are indicated with *vertical lines*. **a** Numerous electron-dense lead deposits in a cell wall of meristematic cell of *A. cepa* root treated with 100 μM Pb(NO_3_)_2_ for 2 h, X-ray spectrum collected over the circled region of the cell wall shows lead characteristic peaks proving the presence of lead in the electron-opaque deposits; **b** large granular deposits in a vacuole of a meristematic cell of *A. cepa* root treated with 100 μM Pb(NO_3_)_2_ for 2 h and X-ray spectrum collected over them proving the presence of lead; **c** compact large deposits in a vacuole of a meristematic cell of *A. cepa* root pre-incubated with 250 μM ATH-rich extract for 3 h and subsequently treated with 100 μM Pb(NO_3_)_2_ for 2 h and X-ray spectrum collected over those precipitates proving the presence of lead; *CW* cell wall, *N* nucleus, *V* vacuole

X-ray microanalysis proved that the electron-dense deposits observed in different cell compartments in the root meristematical cells of Pb(NO_3_)_2_-treated plants not pre-incubated as well as pre-incubated in ATH-rich extract contained Pb (Fig. 7a–c). The compact deposits in vacuoles of the ATH pretreated material contained higher amount of Pb than granular ones in vacuoles of the root meristematic cells treated only with lead (Fig. 7b, c).

## Discussion

Induction of anthocyanin synthesis has been reported in response to different kinds of environmental stressors such as wounding, chilling and especially metal contamination (Gould 2004). It was proved that Cd, Ni, Pb, Zn, Cu and Se enhanced ATH synthesis in many plant species (Dai et al. 2006; Hale et al. 2001; Hawrylak-Nowak 2008; Krupa et al. 1996; Posmyk et al. 2009b). The increase in ATH content was even proposed as a test parameter reflecting the degree of metal toxicity (Hawrylak-Nowak 2008). The observed influence of environmental stresses on the level of anthocyanins in higher plants raised the question whether these common flavonoids can act only as antioxidative factor (Havsteen 2002; Kong et al. 2003) or also alleviate stress by other mechanisms. Numerous authors have shown that flavonoids, to which ATH belongs, are able to form strong ligand complexes with ions of minerals, such as Fe, Cu and Mo causing metal isolation and sequestration (Ferguson 2001; Hale et al. 2001; Havsteen 2002; Rice-Evans et al. 1997).

Sequestration of excess metals in the vacuoles of cells appears to be a common mechanism of metal accumulation and plays a significant role in plant tolerance. Within the cell, metal ions can be chelated by compounds containing –SH groups, e.g. phytochelatins, glutathione, organic acids and other ligands. The role of anthocyanins in metal sequestration was highlighted for the first time by Hale et al. (2001) after the analysis of Mo-treated *Brassica juncea* seedlings.

Taking into account two above-mentioned mechanisms of ATHs actions, i.e. removal of free radicals and compartmentation of toxic metals in vacuoles, these compounds might quite effectively alleviate metal toxic effects in plants (Kalantari and Oloumi 2005). Therefore it was worth checking if exogenously applied ATH could also protect plants against metal toxicity. As we proved earlier, the ATH-rich extract from red cabbage leaves suppressed mitodepressive and turbogenic effects of Cd, Cr and Pb in *A. cepa* L. root meristem (Glińska et al. 2007). Moreover, it alleviated Cu-induced cytological disturbances in *Vicia faba* root meristematic cells as well as in human lymphocytes (Posmyk et al. 2008; 2009a). Cytological tests on plant and animal cells proved that pre-incubation in the ATH extract before Cu stress was most efficient; however, ATH acted effectively even applied after metal stress. It suggests that this extract not only prevents and limits but also heals cytological injuries caused by Cu stress (Posmyk et al. 2009a). By reason of the obvious positive action of the ATH-rich extract from red cabbage leaves on toxic metal-treated material, it was worth examining if that effect was correlated with chelating properties of ATH. In order to do that, we analysed in detail the effect of that extract on Pb localization at morphological, anatomical and ultrastructural levels.

The results of our research have shown that in *A. cepa* plants treated with lead nitrate, Pb was deposited mainly in the root and only a small portion (less than 1 %) of it was transported to shoots during 2 h of incubation. Such distribution of that metal could be explain not only by the short-time treatment but also by very low mobility of lead. Similarly, even after 4 days of exposition to lead nitrate, approximately 93 % of lead taken up by a whole plant are retained in *Pisum sativum* roots (Małecka et al. 2008). Other authors also confirm that roots are the main site of that heavy metal deposition (Antosiewicz and Wierzbicka 1999; Liu et al. 2000; Piechalak et al. 2002, 2003). Pb retention in roots is based on Pb binding to ion exchangeable sites on the cell wall and extracellular precipitation, mainly in the form of Pb carbonate deposited in the cell wall (Inoune et al. 2012; Sharma and Dubey 2005). Once absorbed by roots, Pb is rather immobile, showing very limited translocation into aboveground parts of plants (Kabata-Pendias and Pendias 1999; Piechalak et al. 2003).

The pre-incubation of *A. cepa* roots in the ATH-rich red cabbage extract did not change the total Pb uptake during the short-time treatment with lead nitrate, but it enhanced the translocation of the accumulated metal to shoots. Synthetic chelators such as EDTA, DTPA, NTA and EDDS postulated in chemically assisted phytoremediation are effective in enhancing Pb transport to aboveground parts of plants (Alkorta et al. 2004; Luo et al. 2006; Meers et al. 2005). It has been proposed that addition of EDTA onto soils enhanced translocation of Pb from roots to shoots by lowering its level bound with cell walls (Saifullah et al. 2009). We observed the same mechanism in the case of red cabbage extract.

Examination of rhodizonate-stained cross sections of apical parts of *A. cepa* roots pretreated with ATH clearly demonstrated lack of Pb deposits in the cell walls of outer cortex, while in the material treated only with Pb, that metal was mostly accumulated in the rhizodermis and outer cortex cell walls. Such pattern of distribution is characteristic of the apoplastic transport. Literature data on lead uptake by plants suggest that this metal, after absorption by the root, is predominantly transported by the apoplast (Seregin et al. 2004; Tung and Temple 1996; Wierzbicka 1995). At the first stages of uptake, its presence was detected exclusively in the outer layers of the root, and after some time, it could be detected at the centre of the root (Seregin et al. 2004; Wierzbicka 1995). In *A. cepa*, lead was transported to the successive layers of the root at a rate of one layer per 5 min and reached the centre after 70–85 min (Wierzbicka 1995). Such fast transport of lead across plant tissues was observed also in other terrestrial plants (Wierzbicka 1987a, b, 1995, 1998).

Pb localization at the ultrastructural level was estimated using TEM because the conventional electron microscopy is often used as an adequate technique for analysing the distribution of electron-dense lead deposits in plant cells (Gzyl et al. 1997; Krzesłowska and Woźny 1996; Samardakiewicz et al. 2012; Wierzbicka 1995, 1998; Wierzbicka et al. 2007). It was shown that less than 4 % of lead was lost during chemical preparation of tissues and the redistribution of lead was so insignificant that it did not affect the image seen in TEM (Antosiewicz and Wierzbicka 1999). Moreover, we proved by X-ray microanalysis that the electron-dense deposits observed in the root cells of Pb(NO_3_)_2_-treated *A. cepa* plants contained Pb.

The distribution of lead deposits in the meristematic cells of *A. cepa* roots was similar to that described by other authors (Gzyl et al. 1997; Wierzbicka 1995; Woźny et al. 1982). Great number of lead deposits in cell walls and vacuoles confirms that this metal is neutralised by immobilisation in those structures (Krzesłowska et al. 2010; Wierzbicka 1995, 1998). Lead deposits were noticed predominately in the cell walls, where they were apparently chelated and bound tightly with middle lamellar polysaccharides as it had been reported previously (Antosiewicz and Wierzbicka 1999; Krzesłowska et al. 2009; Tung and Tample 1996; Wierzbicka 1998). Plant cell wall is regarded as one of the main sites for lead deposition and detoxification (Baranowska-Morek and Wierzbicka 2004; Jarvis and Leung 2002; Wierzbicka 1987a, b, 1998; Woźny et al. 1982). The occurrence of lead deposits in dictyosomal vesicles, endoplasmic reticulum or plasmalemma observed in the examined material is also connected with the mechanisms of metal exclusion from cell metabolism (Wierzbicka 1995, 1998). However, significant quantities of lead in the cytoplasm, mitochondria, plastids and nucleus might cause toxic effects within the root such as decrease in mitotic activity and turbogenic effects described earlier under the same experiment conditions (Glińska et al. 2007) that could lead to restriction of plant growth (Liu et al. 2000; Małkowski et al. 1996; Singh et al. 1997).

The ATH-rich extract lowered the number of Pb deposits in intracellular spaces, cell wall and cytoplasm of root meristematic cells as well as in the organelles important to cell metabolism such as the mitochondria, plastids and nucleus. The Pb deposits were preferably localised in vacuoles which were purple-coloured as they contained ATH. The transport of exogenously applied ATH into the vacuoles of *A. cepa* root meristematic cells was investigated and extensively discussed in the earlier paper (Glińska and Gabara 2011). It seems that ATH are transported from the plasmolemma to the vacuole by multivesicular bodies, and there trapped by anthocyanic vacuolar inclusions. Therefore, it is feasible that ATHs facilitate vacuolar sequestration of metal, thereby allowing plants to separate it from vital biochemical processes in other cell compartments. This separation reduces metal cytotoxicity (Glińska et al. 2007) and consequently leads to better plant growth (Hale et al. 2001).

The obtained data allow us to state that the ATH-rich extract from red cabbage leaves acts as a chelating agent enhancing translocation of Pb from roots to shoots by lowering its level bound to a cell wall. Moreover, it sequestrates taken up Pb in the vacuoles and successfully protects cells from its toxic effects.
